# Sensory-Motor Perturbations in Larval Zebrafish (*Danio rerio*) Induced by Exposure to Low Levels of Neuroactive Micropollutants during Development

**DOI:** 10.3390/ijms23168990

**Published:** 2022-08-12

**Authors:** Jason Henry, Yutao Bai, Florian Kreuder, Minna Saaristo, Jan Kaslin, Donald Wlodkowic

**Affiliations:** 1 The Neurotox Lab, School of Science, RMIT University, Melbourne, VIC 3083, Australia; 2 Australian Regenerative Medicine Institute, Monash University, Clayton, VIC 3800, Australia; 3 Environmental Protection Authority Victoria, EPA Science, Macleod, VIC 3085, Australia

**Keywords:** behavior, aquatic, thermotaxis, micropollutant, pharmaceutical

## Abstract

Due to increasing numbers of anthropogenic chemicals with unknown neurotoxic properties, there is an increasing need for a paradigm shift toward rapid and higher throughput behavioral bioassays. In this work, we demonstrate application of a purpose-built high throughput multidimensional behavioral test battery on larval stages of *Danio rerio* (zebrafish) at 5 days post fertilization (dpf). The automated battery comprised of the established spontaneous swimming (SS), simulated predator response (SPR), larval photomotor response (LPR) assays as well as a new thermotaxis (TX) assay. We applied the novel system to characterize environmentally relevant concentrations of emerging pharmaceutical micropollutants including anticonvulsants (gabapentin: 400 ng/L; carbamazepine: 3000 ng/L), inflammatory drugs (ibuprofen: 9800 ng/L), and antidepressants (fluoxetine: 300 ng/L; venlafaxine: 2200 ng/L). The successful integration of the thermal preference assay into a multidimensional behavioral test battery provided means to reveal ibuprofen-induced perturbations of thermal preference behaviors upon exposure during embryogenesis. Moreover, we discovered that photomotor responses in larval stages of fish are also altered by the as yet understudied anticonvulsant gabapentin. Collectively our results demonstrate the utility of high-throughput multidimensional behavioral ecotoxicity test batteries in prioritizing emerging risks associated with neuroactive drugs that can perturb neurodevelopment. Moreover, we showcase the added value of thermotaxis bioassays for preliminary screening of emerging contaminants.

## 1. Introduction

The behavioral phenotype is the highest-level manifestation of integrated neurological functions. Alterations in neurobehavioral traits have been postulated as sensitive and physiologically integrative endpoints to assess the risks associated with toxicants [1,2]. Considering the vast number of anthropogenic chemicals with unknown properties, there is however an increasing need for a paradigm shift towards rapid and higher throughput behavioral bioassays [3,4,5,6]. Surprisingly, only a handful of studies have so far explored rapid multidimensional behavioral tests batteries utilizing a combination of sensorimotor assays, e.g., startle, phototaxis, thermotaxis, and chemotaxis during predictive eco-neurotoxicity testing [3,4,6,7]. Compared to simple and unstimulated locomotory behaviors, such as alterations in spontaneous swimming speed, the analysis of more complex sensorimotor reactions can provide enhanced insights with ecological and neurotoxicological relevance [3,4,8]. There is a broad range of modes of action amongst chemical compounds and thus there is also a broad hierarchy of neurobehavioral endpoints that can be impacted upon exposure [3,4]. The sensory-motor responses are integrated responses to stimuli that include the sensory organs, data transmission, and processing as well as activation of end effector (e.g., motor circuits). The advantage of utilizing multidimensional behavioral testing is analysis of the impact of chemicals on several different sensory-motor responses, such as, e.g., mechanical, chemical, diverse photic or thermal stimuli. Moreover, automation provides better data reproducibility by avoiding user errors and thus greatly minimizing variables [3,4]. Although the available behavioral tests enable detection of subtle alterations in animal responses induced by low levels of toxic factors, alone they generally do not provide sufficient data on elucidating the mechanisms of such changes, nevertheless they may provide valuable screening and clues for making explanatory hypotheses [3,4]. The latter can be further explored based on a growing number of molecular methods that can be applied to studying the mechanistic basis of behavioral changes in aquatic animals [3].

In this regard, there is still a particular paucity of data on wider applications and incorporation of thermotactic behavioral tests in aquatic toxicology [3,6,8]. This is mostly related to a lack of automated analytical systems that could be used for rapid thermal preference behavioral biotests [8]. Thermotaxis is characterized by directional movement toward (positive thermotaxis) or away from (negative thermotaxis) from a region of perceived optimal or noxious temperatures, respectively [9,10]. The analysis of such behaviors is non-trivial and much more complex than simple photic stimulus. This is because stable thermal gradients or defined temperature zones need to be created and spatiotemporally controlled. Recent successful development of effective thermotactic testing using planarian models and zebrafish laboratory provides a compelling outlook for such functional tests [6,7].

The above discussed test strategies are particularly relevant for the rapid prioritization of neuromodulatory pharmaceutical micropollutants and their mixtures [3,4,5]. There is growing evidence highlighting that the increase in consumption of psychoactive, behavior-modifying pharmaceuticals introduces these emerging contaminants to surface waters worldwide. Urban wastewater treatment plants (WWTPs) are the main emitters of such pollutants into freshwater ecosystems [11,12]. Anticonvulsants, antidepressants, and psychostimulants are becoming pervasive micropollutants of urban waters since a majority of existing technologies are inadequate, providing a removal rate of less than 90% for most pharmaceuticals [13,14,15,16].

The impact of emerging neuromodulating drugs on neurobehavioral functions in the hydrosphere is still profoundly understudied, while the existing evidence suggests that chronic exposure can lead to pleiotropic behavioral perturbations in the diverse taxa of aquatic animals [17,18,19,20,21,22,23]. In contrast to a plethora of behavioral studies on antidepressants such as fluoxetine and the anticonvulsant carbamazepine, little is still known about perturbations of gabapentin that is increasingly prevalent in urban aquatic environments [24,25]. A handful of recent reports suggest its potentially adverse impacts on aquatic organisms, including early developmental stages of zebrafish [26,27].

Furthermore, apart from pollutants with established clinically psychoactive modes of action, little is known about exposure to other pharmaceuticals, such as non-steroidal anti-inflammatory drugs (NSAIDs), which can also induce behavioral perturbations in aquatic animals. A handful of ecotoxicity studies in recent years have shown that ibuprofen can indeed induce some effects on animal behavior [28,29,30].

In this work, we for the first time applied a novel and rapid thermotaxis assay integrated into a multidimensional behavioral test battery to characterize environmentally relevant concentrations of emerging pharmaceutical micropollutants including anticonvulsants (gabapentin, carbamazepine), inflammatory drugs (ibuprofen), and antidepressants (fluoxetine; venlafaxine). Collectively our results demonstrate the utility of high-throughput multidimensional behavioral ecotoxicity test batteries in prioritizing emerging risks associated with pharmaceutical micropollutants. Moreover, we showcase the added value of thermotaxis bioassays for preliminary screening of emerging contaminants.

## 2. Results

### 2.1. Defining Zebrafish Thermotactic Responses

**Figure 1:**
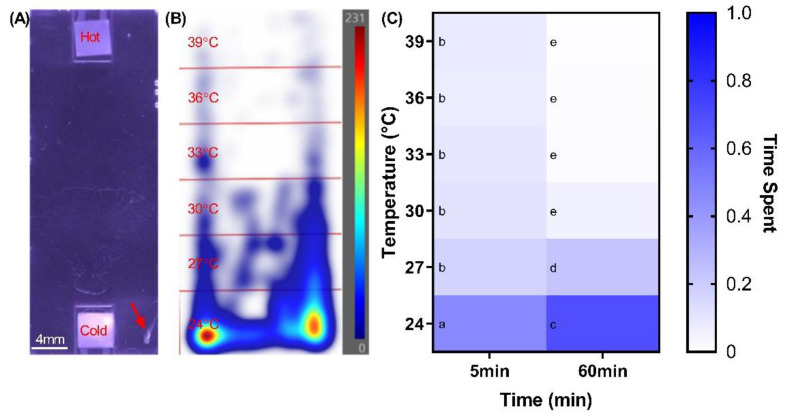
Responses of larval zebrafish to a thermal gradient of 24–39 °C (**A**) Photograph depicting the miniaturized electrothermal array chamber with a zebrafish at 5 dpf, (**B**) An occupancy heatmap of the zebrafish activity highlighting a very strong preference for the 24 °C zone after 60 min of exposure to the thermal gradient (**C**) The thermal preference of zebrafish larvae after 5- and 60-min exposure to the thermal gradient, respectively. Means sharing a letter are not significantly different (*p* > 0.05).

Larval stages of zebrafish exhibit well documented photic as well as thigmotactic and tap startle responses, but there is a paucity of data on their thermotactic behaviors. Accordingly, we set to explore the larval thermotaxis in a stable temperature gradient of 24, 27, 30, 33, 36, and 39 °C generated in a purpose-built, miniaturized electrothermal array system (Figure 1A). Zebrafish at 5 dpf were characterized by a vigorous and very strong thermotactic responses (Figure 1B), with a simple main effects analysis showing that the time spent ratio differed significantly with temperature (F5, 282 = 128.100, *p* < 0.001) in addition to a significant interaction between the effects of zone temperature and immersion time on the time spent in zone ratio (F5, 282 = 10.670, *p* < 0.001). Accordingly, they rapidly migrated to the 24 °C zone after the first 5 min of exposure to the thermal stimulus (0.464 ± 0.053; Mean ± SE) in comparison to the 27 °C (0.171 ± 0.015; Mean ± SE, *p* < 0.001), 30 °C (0.113 ± 0.015; Mean ± SE, *p* < 0.001), 33 °C (0.095 ± 0.017; Mean ± SE, *p* < 0.001), 36 °C (0.072 ± 0.014; Mean ± SE, *p* < 0.001), and 39 °C zones (0.085 ± 0.022; Mean ± SE, *p* < 0.001). Upon 60 min this preference increased to nearly 70% of total time spend in that zone (0.688 ± 0.045; Mean ± SE) (Figure 1C) in comparison to 27 °C (0.229 ± 0.041; Mean ± SE, *p* < 0.001), 30 °C (0.053 ± 0.013; Mean ± SE, *p* < 0.001), 33 °C (0.017 ± 0.005; Mean ± SE, *p* < 0.001), 36 °C (0.008 ± 0.003; Mean ± SE, *p* < 0.001), and 39 °C zones (0.005 ± 0.003; Mean ± SE, *p* < 0.001). The larvae also showed a preference for the 27 °C zone (0.229 ± 0.041; Mean ± SE) compared to the 30 °C (0.053 ± 0.013; Mean ± SE, *p* < 0.001), 33 °C (0.017 ± 0.005; Mean ± SE, *p* < 0.001), 36 °C (0.008 ± 0.003; Mean ± SE, *p* < 0.001), and 39 °C zones (0.005 ± 0.003; Mean ± SE, *p* < 0.001). In summary, zebrafish showed a robust thermotactic response and preference for sub-tropical temperature.

### 2.2. Chemo-Behavioral Alterations upon Exposure to Environmentally Relevant Concentrations of Pharmaceutical Micropollutants

**Figure 2:**
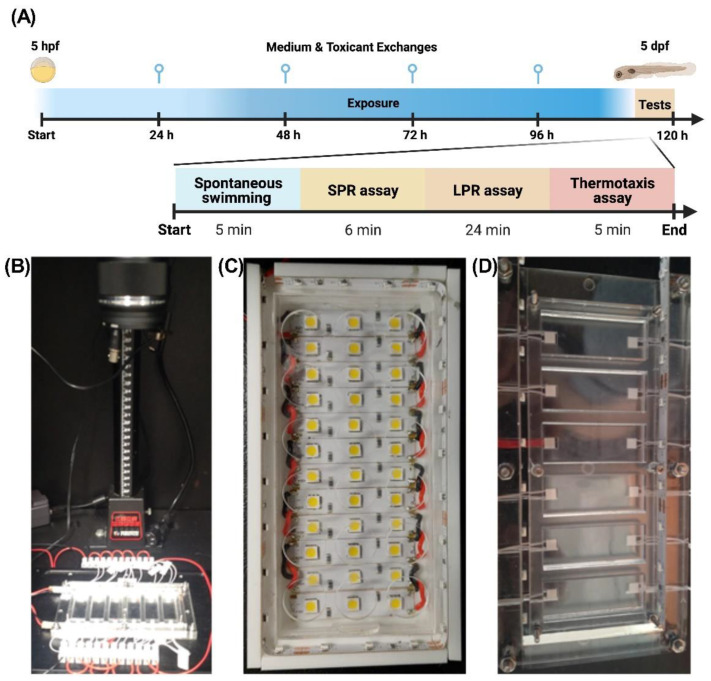
A high throughput multidimensional behavioral test battery (**A**) workflow depicting protocol of the multidimensional behavioral test battery. Zebrafish were exposed to toxicants in the embryonic stage (5 hpf) through to free swimming larval stage (5 dpf), with media exchanges occurring daily. The larval zebrafish then underwent the automated battery comprised of the spontaneous swimming (SS), simulated predator response (SPR), larval photomotor response (LPR) assays as well as a new thermotaxis (TX) biotests; (**B**) Overview of the purpose build analytical system used in the multidimensional behavioral test battery; (**C**) A photic module used for SPR and LPR stimuli biotests, (**D**) An electrothermal module used for thermotactic biotests.

The preliminary data provided strong evidence that robust larval thermotactic behaviors can be successfully employed in ecotoxicity testing. Accordingly, the novel assay was subsequently integrated into a multidimensional behavioral battery consisting of sequential tests, such as spontaneous swimming (SS), simulated predator response (SPR), larval photomotor response (LPR), and a thermal preference (TP) (Figure 2A–D). The test battery was utilized for assessment of environmentally relevant concentrations of emerging pharmaceutical micropollutants detected in urban wastewaters (i) anticonvulsant medications gabapentin (400 ng/L) and carbamazepine (3000 ng/L), (ii) non-steroidal anti-inflammatory drug (NSAID) ibuprofen (9800 ng/L), and (iii) anti-depressants from a selective serotonin reuptake inhibitor (SSRI) class fluoxetine (300 ng/L) and venlafaxine (2200 ng/L) [21,30,31]. The concentrations of chemicals were selected based on empirical data from the recent literature and local sampling conducted by Environmental Protection Authority (EPA) Victoria in 2021 (unpublished). The chosen analytical strategy was aimed to simulate a continuous exposure to pollutants from the earliest stages of ex utero fish embryogenesis till onset of robust independent locomotion and sensory inputs (Figure 2A).

#### 2.2.1. Anticonvulsants

**Figure 3:**
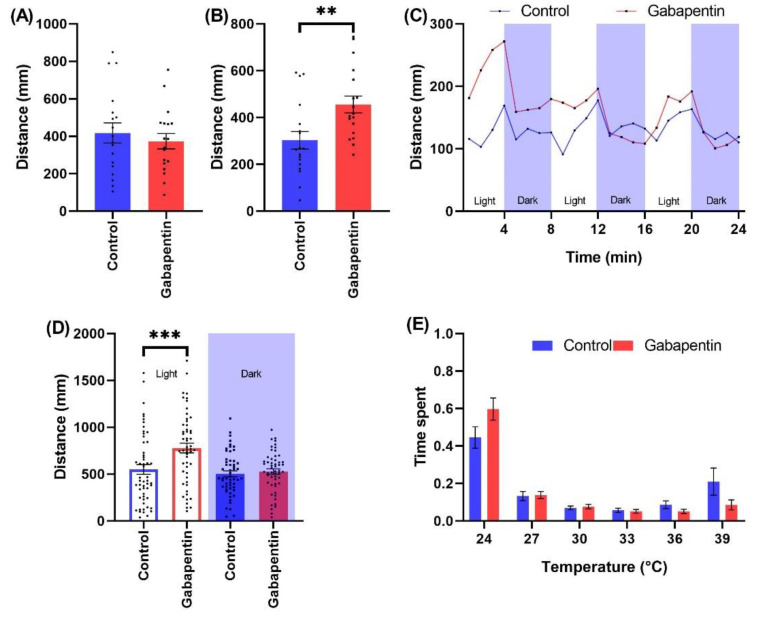
The effects of gabapentin (400 ng/L) exposure on wild type zebrafish behavioral indices during the testing battery. The behavioral assays included (**A**) Spontaneous swimming, (**B**) SPR, (**C**) Time resolved LPR, (**D**) LPR cumulated comparison, and (**E**) Thermotaxis assay. Note ** 0.01 > *p* > 0.001 and *** *p* < 0.001.

Our data demonstrate that neither gabapentin (400 ng/L) nor carbamazepine (3000 ng/L) significantly alter the spontaneous swimming activity of zebrafish larvae at the chosen concentrations of chemicals (Figure 3A and Figure S1A). Individuals exposed to gabapentin were characterized by significantly increased excitation response during the SPR assay (455.539 ± 35.987; Mean ± SE, *p* = 0.006) compared to controls (302.783 ± 37.832; Mean ± SE) (Figure 3B). In the cyclic LPR assay there was a significant overall increase in activity especially during the first two light stimulus phases for gabapentin but not carbamazepine exposed animals (Figure 3C and Figure S1C). Accordingly, the cumulative distance travelled in the light phases of LPR was 778.233 ± 51.851 (Mean ± SE, *p* < 0.001) and 548.418 ± 51.695 (Mean ± SE) for gabapentin and control animals, respectively (Figure 3D). There was no significant effect of gabapentin and carbamazepine on altering the ratio of time spent in any zones of the thermal gradient upon 5 days of exposure (Figure 3E and Figure S1E).

#### 2.2.2. NSAID

**Figure 4:**
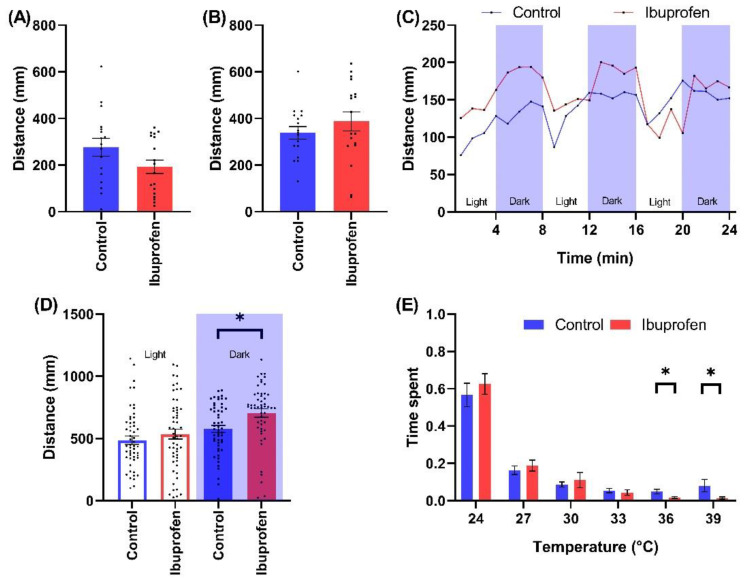
The effects of ibuprofen (9800 ng/L) on wild type zebrafish behaviors throughout the testing battery. The behavioral battery included (**A**) Spontaneous swimming, (**B**) SPR, (**C**) Time resolved LPR, (**D**) LPR cumulated comparison, and (**E**) Thermotaxis assay. Note * 0.05 > *p* > 0.01.

Ibuprofen (9800 ng/L) did not significantly alter the spontaneous swimming activity of zebrafish larvae (Figure 4A). There was also no significant change in excitation response during the SPR assay (Figure 4B). In the cyclic LPR assay there was a significant overall increase in activity especially during dark phases of the first two cycles (Figure 4C). The cumulative distance travelled during all LPR dark phases was 704.603 ± 33.428 (Mean ± SE, *p* = 0.027) and 577.496 ± 28.256 (Mean ± SE) for ibuprofen exposed and control animals, respectively (Figure 4C,D). During the thermal preference test, animals exposed to ibuprofen spent significantly less time in the 36 °C zone (0.018 ± 0.005; Mean ± SE, *p* = 0.035) and the 39 °C zone (0.015 ± 0.005; Mean ± SE, *p* = 0.042) compared to controls (0.048 ± 0.012 and 0.080 ± 0.034, respectively; Mean ± SE) (Figure 4E).

#### 2.2.3. SSRI Antidepressants

**Figure 5:**
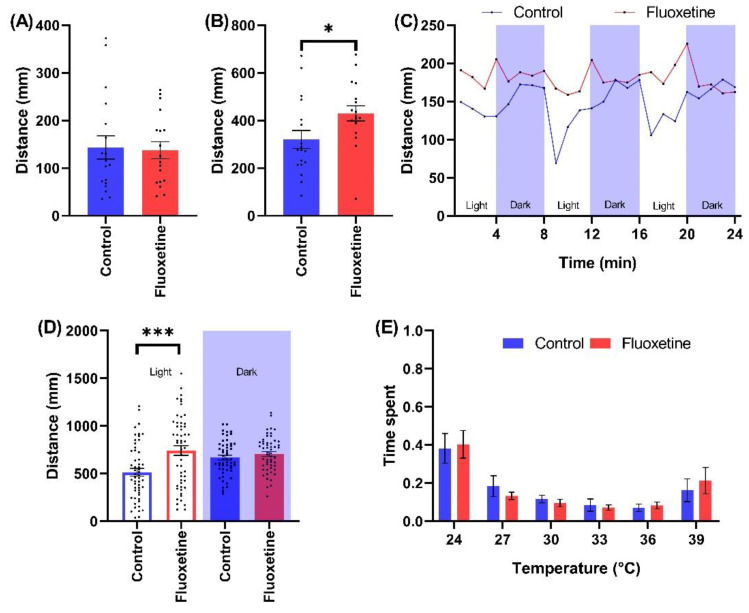
The effects of fluoxetine (300 ng/L) exposure on wild type zebrafish behavioral indices during the testing battery in (**A**) Spontaneous swimming, (**B**) SPR, (**C**) Time resolved LPR, (**D**) LPR cumulated comparison, and (**E**) Thermotaxis assays. Note * 0.05 > *p* > 0.01 and *** *p* < 0.001.

Treatment with fluoxetine (300 ng/L) or venlafaxine (2200 ng/L) did not affect spontaneous swimming activity of zebrafish larvae (Figure 5A and Figure S2A). Fluoxetine exposed individuals were characterized by significantly increased excitation response during the SPR assay (429.961 ± 31.893; Mean ± SE, *p* = 0.034) compared to controls (320.308 ± 38.034; Mean ± SE) (Figure 5B). Both SSRI antidepressants induced a significant increase in mean level behavioral responses during light phases during the LPR assay. This led to a characteristic flattening of the response curve compared to controls (Figure 5C and Figure S2C). The cumulative distance travelled during all LPR light stimulus phases was 741.456 ± 50.970 (Mean ± SE, *p* < 0.001) and 514.266 ± 39.995 (Mean ± SE) for fluoxetine exposed and control animals, respectively (Figure 5D). The same parameter was 936.904 ± 40.558 (Mean ± SE, *p* < 0.001) and 717.421 ± 48.162 (Mean ± SE) for venlafaxine exposed and control animals, respectively (Figure S2D). Neither of the antidepressants impacted the thermal preference responses (Figure 5E and Figure S2E).

## 3. Discussion

### 3.1. Larval Zebrafish Thermotactic Behavior Enables a New Sensory-Motor Biotest

Our preliminary experiments demonstrated that the larval stages of zebrafish exhibited robust thermal preference responses. Given a choice in the thermal gradient they rapidly migrated towards 24–27 °C zones within less than 5 min of exposure. Our data correlates well with a recent study by Gau on heat-induced locomotion of zebrafish larvae [9]. To date, there have been only one recent demonstration of thermotactic behaviors ecotoxicology by Zhang et al. that used Peltier heating plates to induce scrunching behaviors of planarians as well as to analyze planarian thermotaxis [6]. This work included the planarian thermotaxis in a multi-behavioral endpoint testing battery.

Overall, we conclude that thermotactic locomotory avoidance responses are vastly underutilized but represent prospectively added value in ecotoxicity testing [3,6]. In this regard the broad and stable thermal gradient generation devices, such as that presented in this study are much more flexible than conventional shuttle-box systems to explore preference behaviors of very small aquatic model organisms. Moreover, organisms that exhibit strong and rapid thermotaxis, such as zebrafish, are optimal models for inclusion in behavioral ecotoxicity biotests.

The applications of solid-state Peltier systems for much larger chambers/volumes could theoretically be possible but would require a significant re-development of the system and applications of much more powerful Peltier modules. More complex simulations and testing would be required to assure that the balanced and constant temperature of gradient will be maintained through the thermal mass of the liquid within the chamber.

### 3.2. Behavioral Effects upon Exposure to Pharmaceutical Micropollutants during Fish Neuro-Development

#### 3.2.1. Impact of Anticonvulsants

Gabapentin (Neurontin) and carbamazepine (Tegretol) are commonly detected in downstream urban freshwaters at maximum concentrations of up to 400 ng/L and 11,600 ng/L, respectively [14,32]. Our analysis revealed no alterations in spontaneous swimming activity or thermal preference behaviors in hatched zebrafish larvae after exposure to both gabapentin (400 ng/L) and carbamazepine (3000 ng/L) during embryogenesis. The gabapentin exposure induced, however, a significant increase in mean level behavioral responses to photic stimuli, such as increased excitation and an overall increase in activity during the first two light stimulus phases in SPR and LPR assays, respectively. To the best of our knowledge this is the first observation of behavioral perturbations induced by gabapentin at environmentally relevant concentrations. Our data shows that photomotor responses can be altered in larval stages of fish after a reasonably short-term exposure during ex utero embryogenesis.

Reportedly different behavioral phenotypes of carbamazepine and gabapentin can stem from a slightly different mode of action, at least in humans. The carbamazepine is a reported agonist of the gamma-aminobutyric acid (GABA) receptor, and can directly enhance the synaptic transmission [33]. The gabapentin, despite increased use in clinical and veterinary medicine, has a poorly understood mechanism of action [34,35,36]. Considering unclear molecular targets in humans there is a significant scarcity of data on what specific targets the anticonvulsants can affect in for instance aquatic invertebrates, fish, birds, and mammals. The latter aspect is particularly important since in veterinary use gabapentin is known to induce profound behavioral side effects, including excessive sedation and ataxia (incoordination) in some mammals, such as cats [37]. If such effects can also be induced in diverse species of aquatic animals by the excessive exposure to gabapentin it can prospectively lead to profound alterations in ecological survival of particular species.

Overall, we conclude that the potential effects of gabapentin are vastly understudied, but our data together with the existing body of evidence warrants further investigation into this micropollutant, in both short-term and long-term exposure regimens and in mixtures with other psychoactive micropollutants.

#### 3.2.2. Impact of NSAID

Ibuprofen, a non-prescription representative of non-steroidal anti-inflammatory drugs (NSAID), is a widespread micropollutant detected in freshwaters at concentrations of up to 1440 ng/L in Spain and even up to 17,600 ng/L in South Africa [16,38,39,40]. Our data demonstrated that ibuprofen at an environmentally relevant concentration affected the zebrafish visual motor response by inducing an increase in activity during dark phases in the cyclic LPR assay. This observation is consistent with a recent report by Xia et al. that showed reduction of the swimming distance, duration, and speed under dark conditions in larval zebrafish albeit at a concertation two orders of magnitude higher (5 × 105 ng/L) than used in our study [41].

Perhaps most interestingly the embryonic exposure to ibuprofen changed the thermal preference of larval zebrafish since the test animals exposed to ibuprofen spent significantly less time in the 36–39 °C zones. To the best of our knowledge this is the first observation of thermotactic perturbations induced by NSAID at environmentally relevant concentrations.

NSAIDs are known to inhibit prostaglandin synthesis in humans and prostaglandins are involved in thermoregulation [42]. Therefore, NSAIDs drugs can alter body temperature in humans [42]. It is unknown if similar processes occur in non-target aquatic animals or if thermal sensing can be affected by such drugs. This warrants further exploration especially considering potential impact on thermal preference that could affect seasonal migratory patterns between different depths. Furthermore, the anti-inflammatory agents have been shown to significantly change the group behaviors while not significantly affecting individual behaviors in fish [43]. It will thus be important to develop thermal preference assays that could be used in group behavioral experiments with diverse contaminants.

At this stage, it is also not known if the observed thermal preference effects would persist, be accelerated, and result in any long-term behavioral effects at later stages of fish development. To the best of our knowledge this is the first documented example of the aquatic pollutant affecting thermotactic behaviors in larval fish. In fact, the only other reported example of thermotactic test is aquatic ecotoxicology is the work by Zhang et al. that found diazepam can disturb the thermotactic behaviors in planarian *Dugesia japonica* [6]. Our data thus provide therefore further evidence of the need to explore thermal preference behaviors upon exposure to pollutants.

#### 3.2.3. Impact of SSRI Antidepressants

SSRI antidepressants fluoxetine (Prozac) and venlafaxine (Effexor) are detected in freshwaters at concentrations of up to 540 ng/L and 2190 ng/L, respectively [44,45]. Our analysis of environmentally relevant concentrations of fluoxetine (300 ng/L) and venlafaxine (2200 ng/L) demonstrated revealed that both antidepressants induced, a significant increase in mean level behavioral responses to photic stimuli, such as those used in SPR and LPR assays. Overall, the larvae had increased locomotory activity during the light phases compared to untreated animals. The flattening of the response amplitude between light/dark cycles in SSRIs exposed animals reflects the characteristic nature of antidepressants action that homogenize the behavioral responses in humans. Similar trend has recently been observed in guppy fish (*Poecilia reticulata*) where individual variation in population treated with fluoxetine was reduced to half that of controls [20]. Our data show that the identical concentration of fluoxetine used in the work of Polverino et al. can homogenize individuals’ activity and perturb visual motor responses in larval stages of fish after a reasonably short-term 5-day exposure during ex utero embryogenesis. This trend is interesting since it reportedly takes up to 14 days for the treatment with SSRIs to exhibit behavioral effects in humans. Our results are in this regard consistent with a recent work reporting that fluoxetine at environmentally relevant concentrations induces reduction of visual motor response (VMR) in stress-related swimming activity in 107-h old zebrafish embryos [46]. Furthermore, a study by Atzei et al. also reported that both fluoxetine and venlafaxine disturb embryo activity at 96 hpf during the dark periods as well as the light-dark transition in 5 dpf larvae [47].

Interestingly, a recent study by Atzei et al. highlighted another important difference between the two studied antidepressants [47]. The recovery of locomotor functions was observed in larval zebrafish when fluoxetine was removed at 4 dpf but the same exposure scenario for venlafaxine did not lead to any recovery of the behavioral phenotype [47]. This suggests possible developmental neurotoxicity (DNT) effects of that drug. Both drugs have slightly different pharmacological mechanism of action with venlafaxine modulating norepinephrine and dopamine reuptake in addition to serotonin uptake [48,49]. Two recent reports have indeed found that venlafaxine exposure can disrupt brain monoamine levels and neuroendocrine responses to stress in rainbow trout as well as perturb brain development in zebrafish [49,50]. The findings of the latter work are very pertinent since the reduced zebrafish larval activity was correlated with reduced serotonin immunoreactivity and tyrosine hydroxylase-positive cell populations in specific larval brain regions [49]. Furthermore, the co-injection of embryos with serotonin alongside venlafaxine recovered brain serotonin immunoreactivity and tyrosine hydroxylase-positive cell populations, and it rescued the venlafaxine-mediated behavioral phenotype [49].

The reported potential DNT effects of venlafaxine, warrant further exploration since long-term behavioral effects of this antidepressant could be more deleterious than those of the fluoxetine on aquatic fauna.

## 4. Materials and Methods

### 4.1. Biological Specimens

Wild-type zebrafish (*Danio rerio*) strain AB/TU were housed in the AquaCore facility at Monash University (Clayton, VIC, Australia), according to standard operating protocols [51,52]. All experiments on zebrafish were approved by and conducted under the oversight of the Monash University Animal Ethics Committee under ethics approvals ERM14481 and ERM17993.

### 4.2. Chemicals and Materials

Test chemicals venlafaxine, gabapentin, ibuprofen, and carbamazepine were purchased as solid powders from Sapphire-Bioscience (Melbourne, Australia). Fluoxetine was obtained as a stock solution in liquid form, dissolved in methanol, from the Wong lab (Monash University, Melbourne, Australia). Solutions of fluoxetine were prepared through diluting the stock solution directly to the described concentrations. Venlafaxine, ibuprofen, and carbamazepine were dissolved directly in dimethyl sulfoxide (DMSO) to form a stock solution. Stock solutions of gabapentin were dissolved directly in filtered water. DMSO and methanol were purchased from Sigma–Aldrich (Melbourne, Australia). The carbamazepine and gabapentin stock solutions were stored at −20 °C. Subsequent serial dilutions of toxicants were made fresh each day from stock solutions.

Embryo medium E3 was prepared by dissolving 5 mM NaCl, 0.17 mM KCl, 0.33 mM CaCl_2_, 0.33 mM MgSO_4_, 0.1% methylene blue in double-distilled water. The pH was adjusted to 7.8 ± 0.3. Filtered freshwater and seawater were obtained from RMIT University Aquatic Facility.

### 4.3. Chemical Exposure Conditions

All chemical exposures were conducted by placing individual zebrafish embryos at 5-h post fertilization (hpf) stage in individual wells of 48 well plates with 1 mL of exposure solution. Treatment media were replaced with fresh solutions daily. At 5 days post fertilization (5 dpf) behavioral assays were conducted on the hatched zebrafish larval stages. In each experiment, 36 larvae were used per treatment. Vehicle controls were used to account for any potential solvent effects. The impact of solvent concentrations (0.00003% DMSO and 0.0006% methanol by volume) on behavioral indices was considered to be negligible [19,20,21].

### 4.4. Behavioral Biotests

To avoid the influence of circadian rhythm, a behavioral test battery was always performed at the same time of the day for all treatments and chemical compounds. A total of 48 individuals per exposure treatment group were recorded

#### 4.4.1. Spontaneous Swimming (SS) Assay

Tests were conducted in ambient conditions of 25.0 ± 0.5 °C in a temperature-stabilized room. Individual larvae were randomly selected and placed in custom made 18-well PMMA plates with individual chamber dimensions of 15.6 mm in diameter and 3 mm in depth (nominal volume of 570 µL). They were allowed to acclimate in the dark for 2 min and their unstimulated behavior was then video recorded for 5 min. The endpoint for this assay was the swimming activity of the zebrafish represented as the total distance moved (mm) during the entire 5-min period [52].

#### 4.4.2. Simulated Predator Response (SPR) Assay

Specimens remained in the same test plates upon completion of the SS assay. They were allowed to acclimate for 2 min with a uniform white LED backlighting activated (9000 Lux). The behavior was then recorded for 3 min and upon automated switching off the light for the additional 3 min. The endpoint for this assay was the rapid startle locomotor response to sudden dark conditions simulating a passing predator. This endpoint was measured as the total distance moved (mm) during 3 min of dark phase [51].

#### 4.4.3. Larval Photomotor Response (LPR) Assay

Specimens remained in the same test plates upon completion of the SPR assay, the white backlighting stimulus was re-activated, and the zebrafish were allowed to acclimate for 2 min. Locomotor activity was subsequently recorded for a total of 24 min using 3 automated cycles of alternating light (4 min) and dark (4 min) phases. The endpoint for this assay was the cumulated mean locomotor activity of the zebrafish in the light and dark conditions, which was measured as the total distance moved (mm) during this period [52]. The comparative larval dark startle response and light response between treatments was time binned into 1 min increments and averaged for a comparison.

#### 4.4.4. Thermal Preference Assay

Upon completion of the LPR assay, individual specimens were transferred to custom made 6-well PMMA plates with chamber dimensions of 40 × 15 × 4.5 mm and a nominal volume of 2700 µL. The plates were inserted into a purpose-built Peltier array with 12 miniaturized and solid-state thermoelectric cooling/heating elements (ET-007-06-11, Adaptive Thermal Management, Kibworth, UK). Actuation of each pair of thermoelectric elements in opposing cooling and heating regimens allowed for rapid generation a self-balancing and reproducible thermal gradient across each chamber. The Peltier array was sized specifically for thermal mass of the chambers above. The system enabled establishment of six defined temperature regions with a central region mean of 24, 27, 30, 33, 36, and 39 °C along the thermal gradient withing 3 min of operation.

Animals were allowed to acclimate for 2 min, and the behavior was video-recorded for 5 min, following a 3 min thermal gradient establishment period. The behavioral indices were the ratios of time spent in each of the defined 6 temperature regions (central region mean of 24, 27, 30, 33, 36, and 39 °C) along the thermal gradient. This was calculated through defining 6 zones of equal size in Ethovision XT ver 16 animal tracking software and outputting the quantity of time the animal spent in each zone of the arena.

### 4.5. Behavioral Data Acquisition

Behavioral data was acquired using a custom built digital video imaging system consisting of a BlackMagic Micro Studio 4K digital camera (BlackMagic Design, Melbourne, Australia) mounted on a vibration-less photographic column (Polaroid M3, Polaroid Inc., Minnetonka, MN, USA). The camera was custom converted to infrared imaging (IR, 850 nm) and paired with a true 1:1 macro-objective lens with a focal length of 30 mm (Olympus, Tokyo, Japan) [5,51]. For multi-species thermotactic responses videos were recorded at a resolution of 1920 × 1080 pixels (1080p) and a rate of 25 fps. The files were acquired using an external High-Definition Multimedia Interface (HDMI) recorder (Atomos Shogun, Melbourne, Australia) equipped with a programmable time-resolved video acquisition functionality. All remaining assays were recorded at a resolution of 1280 × 720 pixels (720p) and a framerate of 30 fps via a PC HDMI interface (BlackMagic 4K Decklink Mini card, BlackMagic Design, Melbourne, Australia).

### 4.6. Data Analysis and Statistics

Animal tracking was performed using Ethovision XT ver. 16 (Noldus Information Technology, Wageningen, The Netherlands) as described in detail earlier [5,51]. Briefly automatic frame-by-frame tracking produced time-stamped x,y coordinate pairs assigned to centroids of detected objects and provided a foundation for the reconstruction of graphical animal trajectories and behavioral parameters (i.e., average distance travelled, time spent in arena zone) calculated for each test subject. Numerical data sets were exported to Excel files. Subsequent visualization and statistical analysis were performed in Prism 9 software (GraphPad, San Diego, CA, USA).

All data were first checked for normality with Shapiro–Wilk and Kolmogorov–Smirnov tests. Data found to not be normally distributed was logarithmically transformed to stabilize the variance. A one-way ANOVA with Dunnett’s T3 multiple comparisons test was used to determine statistical significance in assays comparing more than two groups. A two-way repeated ANOVA with Tukey’s multiple comparisons test was used to determine statistical significance in the initial time course zebrafish experiment comparing more than two groups. Comparisons between two groups were analyzed with a standard t-test. In all experiments, data were considered significant when *p* < 0.05.

## 5. Conclusions

In this work, we have demonstrated an innovative, purpose-built system for high throughput multidimensional behavioral test battery on larval stages of zebrafish. We also showed that zebrafish larvae exhibit rapid thermotactic behaviors that can be exploited for enhancing the ecotoxicity screening. In particular, the data indicates the utility of using multidimensional behavioral test batteries for preliminary screening of emerging contaminants [3]. Such sensory-motor test batteries could in the future combine startle stimuli (photic and tap), phototactic (light/dark response and spectral preference), thermotactic, and even chemotactic testing. The photic and tap could be easily implemented in the future using custom LED printed circuit boards (PCBs) and standard solenoids connected into the existing control system. Further to these alterations, differing chambers in terms of size and materials could be used to allow testing on larger organisms, such as adult zebrafish. So far, very little effort has been made to develop and to validate such approaches in predictive neurotoxicology though such testing has already been demonstrated as very impactful in neuroactive drug discovery [3,53,54]. The lack of user-friendly and off-the-shelf analytical technologies capable of facilitating such testing is one of the reasons behind the paucity of data on this emerging topic. Only recently an automated multi-behavioral test battery that included thermotactic assays has been showcased to ecotoxicity screening using a planarian model [6].

The future development of the presented technology could also be considered for terrestrial organisms, such as for instance non-flying insects, albeit several major changes in the design would be required. The constant temperature of gradient of the organism chamber in the current design is maintained through the thermal mass of the liquid within, allowing an interaction between the cooling and heating Peltier modules. To establish the thermal gradient for terrestrial applications the thermal mass of water would need to be replaced with another conductive material.

From a perspective of eco-neurotoxicology the integration of diverse sensory-motor assays in biotest batteries especially when used with genetically trackable model organisms, such as zebrafish, can prospectively allow us to gain a much broader as well as deeper understanding of the impact of environmental toxicants on CNS. Such approaches can also form a foundation of rapid prioritization pipelines for selecting pollutants with neurotoxic and neuromodulating properties for further advanced but also much more time-consuming behavioral tests with fish and invertebrates.

## Data Availability

The datasets generated and/or analyzed in the present study are available from the corresponding author upon reasonable request.

## Supplementary Materials

The following supporting information can be downloaded at: https://www.mdpi.com/article/10.3390/ijms23168990/s1.
