# Supplementary material for: Sensory-Motor Perturbations in Larval Zebrafish (Danio rerio) Induced by Exposure to Low Levels of Neuroactive Micropollutants during Development
Source: Int J Mol Sci. 2022 Aug 12;23(16):8990. doi: 10.3390/ijms23168990 (PMC9409309; doi:10.3390/ijms23168990)
Supplement: Supplementary file 1 [file ijms-23-08990-s001.zip › ijms-1858830-supplementary.pdf]

Electronic supplementary data

Sensory-Motor Perturbations in Larval Zebrafish (*Danio rerio*) Induced by Exposure to Low Levels of Neuroactive Micropollutants during Development

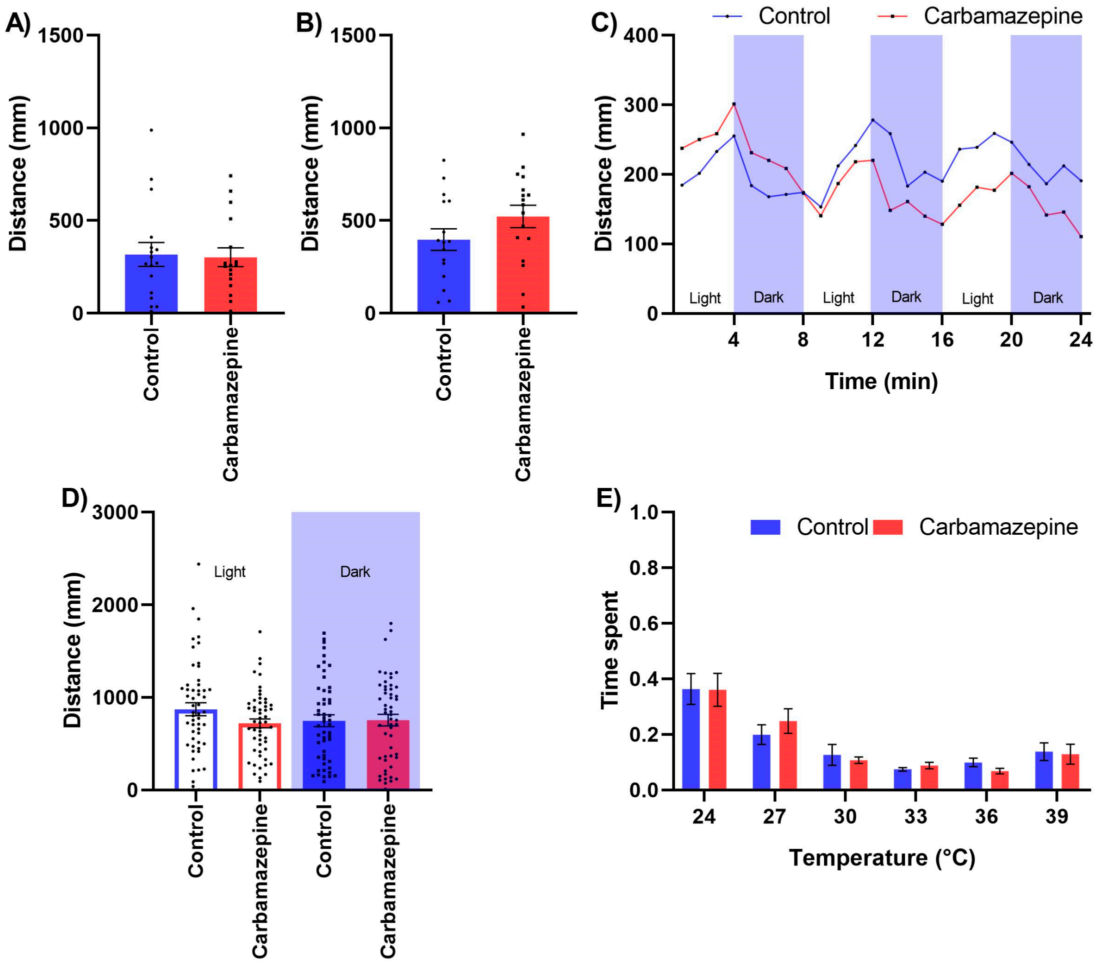

Figure S1. The effects of carbamazepine (3000 ng/L) on wild type zebrafish behaviours during the testing battery. The behavioural battery included (**A**) Spontaneous swimming, (**B**) SPR, (**C**) Time resolved LPR, (**D**) LPR cumulated comparison and (**E**) Thermotaxis assay. Note * 0.05 > p > 0.01, ** 0.01 > p > 0.001 and *** p < 0.001.

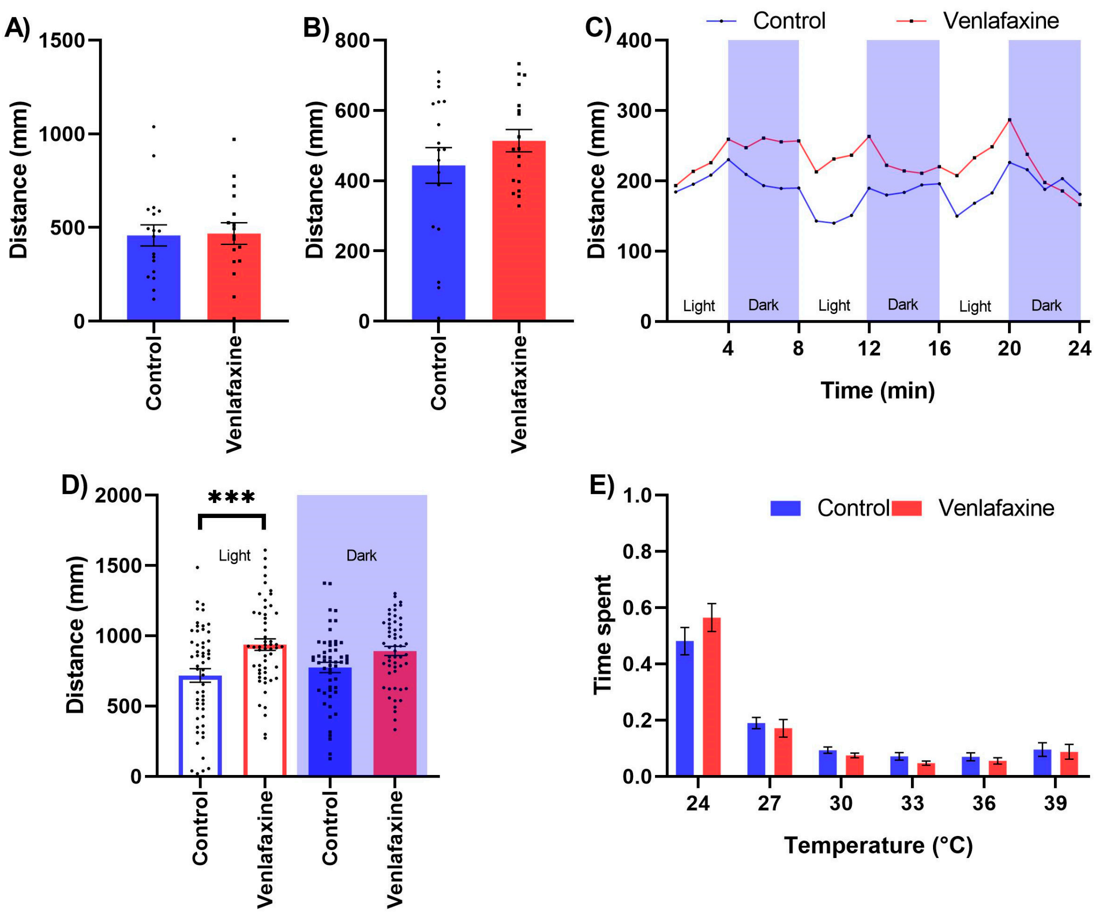

Figure S2. The effects of venlafaxine (2200 ng/L) on wild type zebrafish behaviours undergoing the testing battery. The behavioural battery included (**A**) Spontaneous swimming, (**B**) SPR, (**C**) Time resolved LPR, (**D**) LPR cumulated comparison and (**E**) Thermotaxis assay. Note * $0.05 > p > 0.01$, ** $0.01 > p > 0.001$ and *** $p < 0.001$.
